# Racial Disparities in Brachytherapy Treatment among Women with Cervical and Endometrial Cancer in the United States

**DOI:** 10.3390/cancers15092571

**Published:** 2023-04-30

**Authors:** Kekoa Taparra, Brandon I. Ing, Agnes Ewongwo, Jacqueline B. Vo, Jaimie Z. Shing, Megan Y. Gimmen, Kiana M. K. Keli‘i, Jason Uilelea, Erqi Pollom, Elizabeth Kidd

**Affiliations:** 1 Department of Radiation Oncology, Stanford Health Care, Stanford, CA 94305, USA; 2 Department of Obstetrics and Gynecology, Kaiser Permanente, Los Angeles, CA 90027, USA; 3 Division of Cancer Epidemiology and Genetics, National Cancer Institute, Rockville, MD 20850, USA; 4 Harvard Medical School, Boston, MA 02115, USA; 5 Brown University, Providence, RI 02912, USA

**Keywords:** radial disparities, cancer disparities, endometrial cancer, cervical cancer, brachytherapy, radiation therapy, Native Hawaiian and other Pacific Islanders

## Abstract

**Simple Summary:**

Brachytherapy remains an essential part of the treatment paradigm for women diagnosed with cervical and endometrial cancers. Recent evidence suggests that treatment practice patterns are changing with decline in the use of brachytherapy for patients with cervical cancer. There has yet to be a national study of brachytherapy treatment practice patterns investigating existing disparities among all five of the United States’ federally recognized racial groups. The authors aim to identify racial differences among women with cervical and endometrial cancers among five federally defined United States race categories. Our findings unmasked that Native Hawaiian and other Pacific Islander women with endometrial cancer and Black women with cervical cancer are significantly less likely to receive brachytherapy treatment, particularly those at community cancer hospitals.

**Abstract:**

Brachytherapy improves clinical outcomes among women diagnosed with cervical and endometrial cancers. Recent evidence demonstrates that declining brachytherapy boosts for women with cervical cancer were associated with higher mortality. In this retrospective cohort study, women diagnosed with endometrial or cervical cancer in the United States between 2004 and 2017 were selected from the National Cancer Database for evaluation. Women ≥18 years of age were included for high intermediate risk (PORTEC-2 and GOG-99 definition) or FIGO Stage II-IVA endometrial cancers and FIGO Stage IA-IVA—non-surgically treated cervical cancers. The aims were to (1) evaluate brachytherapy treatment practice patterns for cervical and endometrial cancers in the United States; (2) calculate rates of brachytherapy treatment by race; and (3) determine factors associated with not receiving brachytherapy. Treatment practice patterns were evaluated over time and by race. Multivariable logistic regression assessed predictors of brachytherapy. The data show increasing rates of brachytherapy for endometrial cancers. Compared to non-Hispanic White women; Native Hawaiian and other Pacific Islander (NHPI) women with endometrial cancer and Black women with cervical cancer were significantly less likely to receive brachytherapy. For both NHPI and Black women, treatment at community cancer centers was associated with a decreased likelihood of brachytherapy. The data suggest racial disparities among Black women with cervical cancer and NHPI women with endometrial cancer and emphasize an unmet need for brachytherapy access within community hospitals.

## 1. Introduction

Uterine cancers are among the most common malignancies of women in the United States with annual new case estimates of 66,000 for uterine corpus (endometrial) and 14,000 for uterine cervix (cervical) cancers [1]. Radiation therapy (RT) has played a critical role in the treatment paradigm for gynecological malignancies over the past century [2]. Brachytherapy with or without external beam radiation therapy (EBRT) now serves as a critical component of the curative management of endometrial and cervical cancers [3,4]. In the setting of high intermediate risk endometrial cancer, data from PORTEC-2 shows similar efficacy between brachytherapy and adjuvant EBRT in reducing locoregional recurrence, with reduced GI toxicity [5], whereas for FIGO stage IB2-IVA cervical cancer (disease ranging from deeply invasive to locally metastatic), brachytherapy with EBRT and concurrent chemotherapy demonstrated a survival benefit in large retrospective studies when compared to EBRT boosts [6]. Failure to include brachytherapy in the treatment regimen, particularly within an 8-week interval of diagnosis, is associated with decreased overall survival (OS) [7,8,9].

Among patients from racial minority groups diagnosed with gynecologic malignancies, limited access to standard of care treatments has been a leading contributor to increased morbidity and decreased OS [10]. This has been well-studied among Black women who experience disparities in time to treatment and treatment modalities for gynecologic malignancies, including patients who require brachytherapy treatment [9,11,12,13,14,15]. When compared to White and Black women, prior studies have found that the aggregated Asian and NHPI population had a lower cancer incidence, superior OS, and higher likelihood of receiving guideline-concordant care [11,16,17,18,19,20]. These studies did not disaggregate NHPI patients from Asian patients, as federally defined since 1997 [21], potentially masking existing differences between Asian and NHPI populations [22,23]. To our knowledge, there are no studies that include all five United States racial categories (White, Black or African American [Black], Asian, American Indian or Alaska Native [AI/AN], and Native Hawaiian or other Pacific Islander [NHPI]) investigating brachytherapy treatment practice patterns for endometrial and cervical cancers [21].

Cancer remains the leading cause of death among NHPI women, with rates higher than any other racial or ethnic group in the United States, contrasting from White and Black women with heart disease as their leading cause of death [24]. Nonetheless, the NHPI race is seldom reported in the medical literature [23,25]. The majority of investigations either inappropriately aggregate NHPI patients with Asian patients, or exclude them altogether [25]. Prior studies have shown, when disaggregated according to federal standards, NHPI women with endometrial cancer have inferior survival outcomes compared to Asian and Non-Hispanic White women [26]. There is an unmet need to better understand NHPI cancer disparities using data disaggregation in compliance with federal race categories. Therefore, the purpose of this study was to quantify the differences in brachytherapy use among women with cervical cancer or endometrial cancer across all five federally recognized race groups. The specific objectives of this study were to (1) outline treatment practice patterns for brachytherapy by patient race and year of cancer diagnosis, (2) identify differences in rates of brachytherapy treatment by racial groups, and (3) determine associated predictive factors among populations significantly less likely to receive brachytherapy.

## 2. Materials and Methods

### 2.1. Study Design

This retrospective cohort study was exempt from Stanford University Institutional Review Board review given the deidentified and publicly available data used. The National Cancer Database (NCDB) is a United States hospital-based dataset that includes >70% of all newly diagnosed cancers in the United States [27]. 

### 2.2. Study Population

Women with diagnostically confirmed stage IA-IVB cervical or endometrial cancer, age ≥18 years, and with ≥12 months follow-up, were included for analysis. Women who were most likely to benefit from brachytherapy, compared to EBRT, for local control with reduced treatment toxicity, were included in this study, in alignment with previous clinical trial inclusion criteria. For example, women with endometrial cancer were included if they underwent definitive surgical resection, met the inclusion criteria for high intermediate risk disease as defined by either PORTEC-2 [5] or GOG-99 [28] definitions, or were diagnosed with FIGO stage II-IVA endometrial cancer. PORTEC-2 inclusion criteria for high intermediate risk were women with FIGO stage I-IIA endometrial cancer who met one of the following criteria: women ≥60 years of age with lower-grade 1–2 tumors and ≥50% myometrial invasion; or women age ≥60 with higher-grade 2–3 and <50% myometrial invasion; or endocervical glandular involvement [5]. The GOG-99 definition of high intermediate risk was defined as women age ≥70 years with at least one risk factor, women age 50–69 years with two risk factors, and women with all three risk factors: high grade 2–3, deep myometrial invasion (FIGO Stage IB), and presence of lymphovascular space invasion (LVSI) [28]. Women with cervical cancer who underwent surgical treatment were excluded from the study, given brachytherapy is not routinely used in this setting. Patients were also excluded if they had any missing survival, staging, or treatment (surgery, RT, or chemotherapy) data, considering that this information was needed to select patients who would optimally benefit from adjuvant RT. All race categories assessed were classified as per United States Office of Management and Budget 1997 federal standards: White (Non-Hispanic; majority reference group), Black, Asian, AI/AN, and NHPI. Patients missing this data were excluded as it was a primary covariable of interest.

### 2.3. Outcomes and Covariable Definitions

The primary outcome of the study was rates of RT modality treatment (brachytherapy, EBRT, or combined brachytherapy and EBRT). Days to RT was defined by the interval of time between the date of diagnosis and the start of RT. OS was defined as the number of months from initial cancer diagnosis to the last follow-up or death. Patient-level characteristics included age at diagnosis (years), residential distance from hospital (per 100 miles), income (above or below median population household income), rurality (urban/rural or metropolitan), education (above or below median percentage of population without a high school degree), insurance status (private, Medicaid/Medicare, or uninsured), comorbidities status (Charlson Deyo Comorbidity score of 0–2 or ≥3), facility type (academic/research program, community cancer program, comprehensive community cancer program, or integrated network cancer program—as defined by the Commission on Cancer designations [27]), year of diagnosis (2004–2010 vs. 2011–2017), and United States region (West, Midwest, Northeast, and South). The variables for socioeconomic status were estimated with zip-code or centroid based census-level data, as defined by the NCDB [27]. Additional cancer characteristic variables included cancer grade, stage, histology, and LVSI.

### 2.4. Statistical Analysis

Descriptive statistics were reported as frequency (%) and median (interquartile range (IQR)) for categorical and continuous variables, respectively. Fisher’s exact and ANOVA tests were used to compare categorical and continuous variables, respectively. OS was assessed using Kaplan–Meier (KM) estimates and log-rank tests. Multivariable logistic regression estimated adjusted odds ratios (aORs) of undergoing brachytherapy for each race compared to White patients, after adjusting for age, year of diagnosis, comorbidity burden, distance from hospital, education, income, rurality, insurance status, facility type, facility region, chemotherapy, and cancer grade. Sensitivity analyses were performed to evaluate how missingness of data potentially impacted the results of the logistic regression analyses. Among races with significantly lower likelihood of receiving brachytherapy compared to White women, multivariable logistic regression models identified significant predictors of receiving brachytherapy, adjusting for the aforementioned characteristics. The 95% confidence intervals (95% CI) were calculated for each corresponding statistic. Cochran–Armitage trend tests were used to assess statistical trends in patients who were treated with brachytherapy ± EBRT versus EBRT alone across year of cancer diagnosis, stratified by cancer type. Tests were two-tailed, with a significant *p*-value threshold of 0.05. All statistical tests were performed using R v4.0.3 in RStudio 2022.12.0+353 (Boston, MA, USA).

## 3. Results

### 3.1. Patient Demographics and Cancer Characteristics

**Figure 1:**
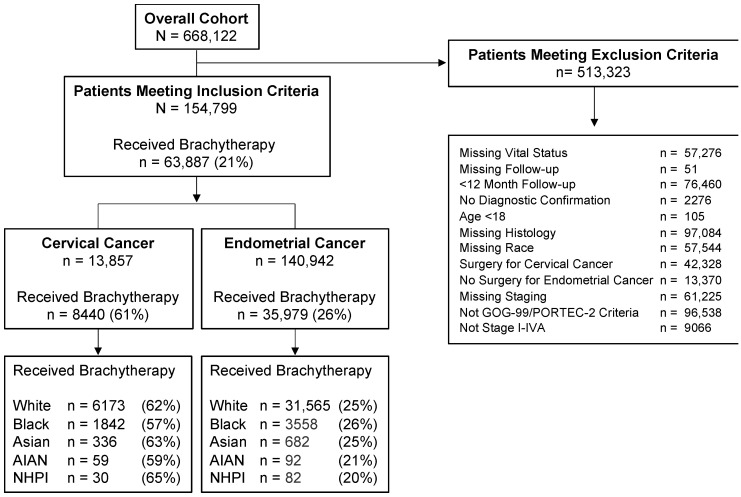
The study flow diagram depicts the number of patients who met inclusion and exclusion criteria. The overall cohort eligible for analysis was stratified by cancer (cervical cancer versus endometrial cancer), radiation treatment type (brachytherapy versus no brachytherapy), and race. The percentage calculated shows the percent of patients who underwent brachytherapy compared within each group. Brachytherapy includes patients who underwent brachytherapy either alone or in combination with external beam radiation therapy. Abbreviations: White = Non-Hispanic White; AI/AN = American Indian and Alaska Native; NHPI = Native Hawaiian and Other Pacific Islander.

Figure 1 shows the study flow diagram detailing the patients included and excluded from the study. Of the 668,122 patients in the initial cohort with cervical and endometrial cancer available to assess, 154,799 patients met inclusion criteria (Table 1). Of those women who met the inclusion criteria, 13,857 women with cervical cancer and 140,942 women with endometrial cancers were included. 

Table 1 shows the patient characteristics of the study. Women with endometrial cancer had a median age of 64 years and a median follow up time of 71 months. Women with cervical cancer had a median age of 53 years and median follow-up time of 45 months. Table 1 shows women with cervical cancer in the study were predominantly White (72%), with lower income (50%), from metropolitan areas (78%), with less education (53%), with a comorbidity index of ≤2 (96%), were treated at academic centers (42%), and were treated in facilities in the South (34%). Table 1 shows women with endometrial cancer in the study were predominantly White (88%), with higher income (56%), from metropolitan areas (81%), with more education (55%), with a comorbidity index ≤2 (95%), were treated at academic centers (40%), and were treated in the South (35%). The most common cancer grade was Grade 2 for both cervical (50%) and endometrial (45%) cancers. Stage III was most common for cervical (44%), whereas stage IA/IB was most common for endometrial cancer (72%). Overall mortality rate was 44% for all women with cervical cancer and 24% for all women with endometrial cancer.

### 3.2. Unadjusted Cancer Treatment Practice Patterns by Race

Table 2 and Table 3 show the cancer treatment practice patterns stratified by the five federally defined United States race categories for women with cervical and endometrial cancer, respectively. Generally, there was a range of rates for treatment practice patterns for RT among women with cervical and endometrial cancer (Table 2 and Table 3). For the entire cohort, 21% of patients received any brachytherapy for treatment (Figure 1). Stratified by cancer, 61% of women with cervical cancer who met inclusion criteria received any brachytherapy (Figure 1; Table 2) while 26% of women with endometrial cancer who met inclusion criteria received any brachytherapy (Figure 1; Table 3). 

Unadjusted for other factors, NHPI women with cervical cancer received brachytherapy at proportionally the highest rates (65%; Figure 1; Table 2), whereas Black women with cervical cancer received brachytherapy at proportionally the lowest rates (57%). Conversely, Black women with endometrial cancer received brachytherapy at proportionally the highest rates (26%; Figure 1; Table 3), whereas NHPI women with endometrial cancer received brachytherapy at proportionally the lowest rates (20%). 

Among women with cervical cancer, there were differences in radiation therapy and chemotherapy treatment practice patterns (Table 2). Black women with cervical cancer had proportionally the highest rates of treatment with brachytherapy alone (16%). NHPI women with cervical cancer had the highest rates of any brachytherapy treatment alone or in combination with EBRT (65%). There was little variation in the time from diagnosis to RT by race (overall cohort median 37 days; IQR = 23–56 days). Most women with cervical cancer (86%) underwent chemotherapy as a part of their treatment. 

Among women with endometrial cancer, there were observed differences in RT and chemotherapy treatment practice patterns (Table 3). Black and White women with endometrial cancer had the highest rates of brachytherapy alone (18%) or in combination (26%). Black women were proportionally the most likely to receive chemotherapy (28%). The median time from diagnosis to radiation treatment among patients with endometrial cancer varied by race: 92 days for White women, 95 days for AI/AN women, 102 days for Asian women, 104 days for NHPI women, and 109 days for Black women.

### 3.3. Radiation Trends and Overall Survival

**Figure 2:**
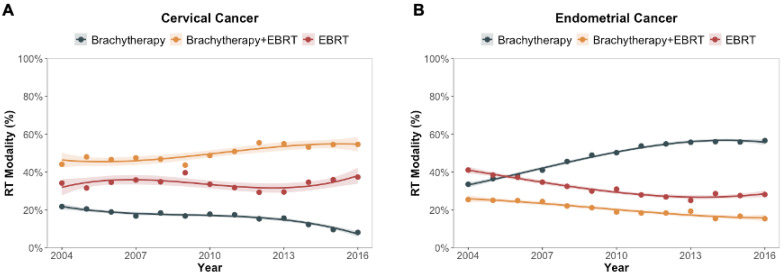
Changes in practice patterns among women who received radiation therapy for treatment of cervical cancer (**A**) and endometrial cancer (**B**) in the United States between 2004 and 2016. The year represents the year of cancer diagnosis. The shaded regions represent the 95% confidence intervals. Abbreviations: EBRT = external beam radiation therapy; RT = radiation therapy.

Figure 2 displays the changes in proportion of RT by radiation modality over time (year of cancer diagnosis) for women with cervical cancer (Figure 2A) and endometrial cancer (Figure 2B). For women with cervical cancer treated with RT, the rate of treatment with brachytherapy ± EBRT (Figure 2A; orange and black lines) was 66% (576 of 875 women) in 2004 and 63% (786 of 1254 women) in 2016. There was not a significant trend by year of diagnosis among women with cervical cancer treated with RT when specifically comparing those treated with brachytherapy ± EBRT versus EBRT alone using the Cochran–Armitage trend test (*p* = 0.996). For women with endometrial cancer treated with RT, the rate of treatment with brachytherapy ± EBRT (Figure 2B; orange and black lines) was 59% (1679 of 2848 women) in 2004 and 72% (3475 of 4832 women) in 2016. There was not a significant trend by year of diagnosis among women with cervical cancer treated with RT when specifically comparing those treated with brachytherapy ± EBRT versus EBRT alone using the Cochran–Armitage trend test (*p* = 0.739). 

**Figure 3:**
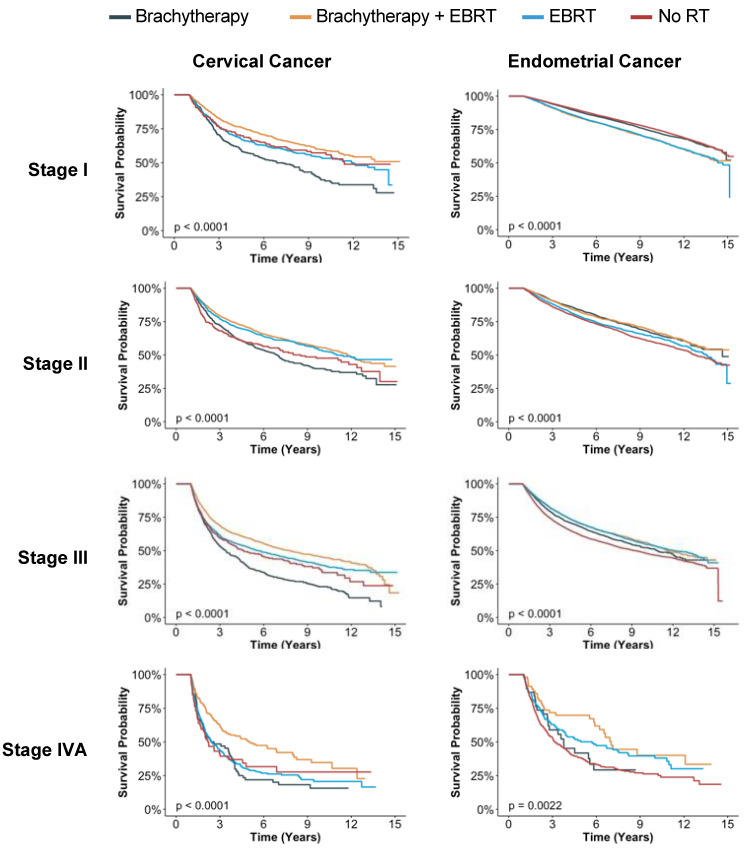
Kaplan–Meier analyses for overall survival stratified by cancer type, cancer stage, and radiation treatment modality. Statistical significance was assessed with log-rank test. Abbreviations: EBRT = external beam therapy; RT = radiation therapy.

Figure 3 shows OS of women with cervical and endometrial cancer treated with RT stratified by radiation modality (brachytherapy alone, EBRT alone, combined brachytherapy and EBRT, and no RT). Each plot is stratified by stage I–IVA. There were significant differences in the OS for women receiving different RT treatment modalities among all groups when stratified by cancer and stage with *p* < 0.0001 for all analyses except for stage IVA endometrial cancer (*p* = 0.0022). For women with stage I and III cervical cancer, brachytherapy alone had the worse OS. Across all stages of cervical cancer, combined brachytherapy and EBRT appeared to have the best survival (orange line). For patients with stage II–IVA endometrial cancer, patients who did not receive any radiation generally appeared to have the worse survival. For women with stage III endometrial cancer, no RT appeared to have worse OS. For women with stage IV endometrial cancer, no RT or brachytherapy alone appeared to have worse OS.

### 3.4. Racial Disparities and Predictors of Not Receiving Brachytherapy

**Figure 4:**
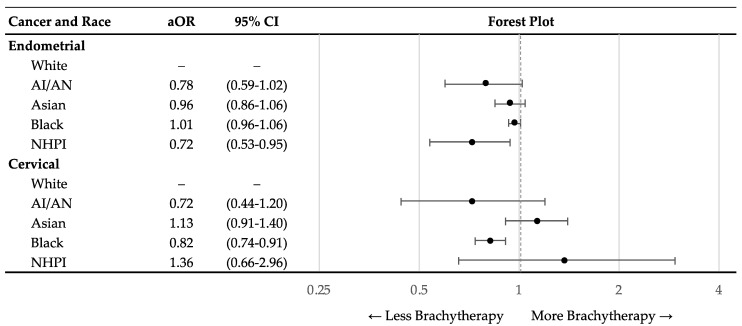
Adjusted multivariable logistic regression analysis of race as a predictor for patients who receive brachytherapy. The analysis was stratified by cancer type and by race. The forest plot displays adjusted odds ratios with 95% confidence intervals. The multivariable regression was adjusted for age, year of diagnosis, comorbidity burden, distance from hospital, education, income, rurality, insurance status, facility type, facility region, treatment with chemotherapy, cancer stage, histology, and cancer grade. The dashed gray line represents an adjusted odds ratio of 1. Abbreviations: White = Non-Hispanic White; AI/AN = American Indian and Alaska Native; NHPI = Native Hawaiian and Other Pacific Islander; aOR = adjusted odds ratio; 95%CI = 95% confidence interval.

Multivariable logistic regression models were performed to predict which race, if any, was associated with not receiving brachytherapy stratified by cancer site. Figure 4 shows the results of the logistic regression models with corresponding forest plot. The models were adjusted for age, year of diagnosis, comorbidity burden, distance from hospital, education, income, rurality, insurance status, facility type, facility region, treatment with chemotherapy, cancer stage, histology, and cancer grade. Black women with cervical cancer had significantly lower likelihood of being treated with any brachytherapy (aOR = 0.82; 95% CI = 0.74–0.91) compared to White women. NHPI women with endometrial cancer had significantly lower likelihood of being treated with any brachytherapy (aOR = 0.72; 95% CI = 0.53–0.95) compared to White women. Sensitivity analyses did not reveal differences in these results based on the inclusion of patients who were originally excluded for missing data.

**Figure 5:**
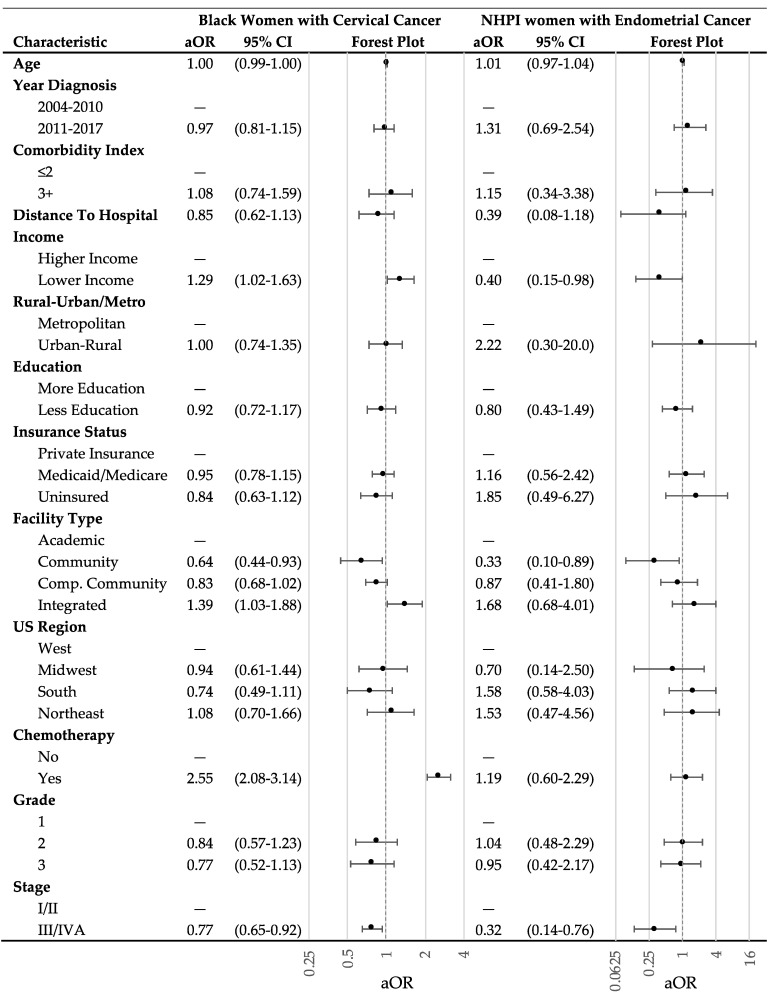
Multivariable logistic regression models for patient predictors of receiving brachytherapy among Black and NHPI women with endometrial cancer. Both analyses were adjusted for patient age, year of diagnosis, comorbidity index, distance to hospital (per 100 miles), income, rurality, education, insurance, facility type, facility region, chemotherapy, cancer grade, and cancer stage. The dashed gray line represents an adjusted odds ratio of 1. Abbreviations: White = Non-Hispanic White; AI/AN = American Indian and Alaska Native; NHPI = Native Hawaiian and Other Pacific Islander; academic = academic/research program; community = community cancer program; comp. community = comprehensive community cancer program; integrated = integrated network cancer program; aOR = adjusted odds ratio; 95% CI = 95% confidence interval.

To investigate predictors of receiving brachytherapy among populations that had significantly lower likelihood of receiving brachytherapy, multivariable logistic regression models were performed, stratified by race, displayed with corresponding forest plots (Figure 5). Black women with cervical cancer and NHPI women with endometrial cancer were significantly less likely to receive brachytherapy when treated at community cancer centers (Black women: aOR = 0.64, 95% CI = 0.44–0.93; NHPI women: aOR = 0.33, 95% CI = 0.10–0.89; compared to women treated at academic centers). Both Black women with cervical cancer and NHPI women with endometrial cancer were also less likely to receive brachytherapy when diagnosed with stage III/IVA cancers (Black women: aOR = 0.77, 95% CI = 0.65–0.92; NHPI women: aOR = 0.32, 95% CI = 0.14–0.76; compared to stage I/II cancers). Specifically for Black women with cervical cancer, treatment with brachytherapy was associated with lower income (aOR = 1.29, 95% CI = 1.02–1.63; compared to higher income) and treatment integrated network cancer centers (aOR = 1.39, 95% CI = 1.03–1.88; compared to women treated at academic centers). Among Black women with cervical cancer, treatment with chemotherapy was associated with an increased likelihood of receiving brachytherapy (aOR = 2.55, 95% CI = 2.08–3.14; compared to no chemotherapy).

## 4. Discussion

In this large, hospital-based study of over 13,000 women with cervical cancer and over 140,000 women with endometrial cancer, we identify treatment practice pattern variation between races. To our knowledge, this is the first large scale national United States analysis that properly includes and disaggregates all five racial categories, including NHPI women. The data reveal NHPI women with endometrial cancer had proportionally the lowest rates of treatment with any form of brachytherapy for endometrial cancer, whereas Black women had the lowest proportion for cervical cancer. However, there were lower rates of brachytherapy use for endometrial cancers, compared to cervical cancers, across races. Adjusted for patient demographics, cancer characteristics, and treatment modalities, NHPI women with endometrial cancer and Black women with cervical cancer had significantly lower likelihood of being treated with brachytherapy, compared to non-Hispanic White women, and both were less likely to receive brachytherapy treatment when treated at community cancer centers. This study underscores the importance of the inclusion of all five races and proper data disaggregation, particularly among the NHPI population. Despite experiencing disproportionately high cancer disparities, NHPI patients are known to be frequently excluded from the medical literature, thus masking significant cancer disparities [22,23,25,29]. 

This study highlights brachytherapy disparities is in the context of recent clinical trials that established current standard of care for women with gynecological malignancies. PORTEC-1 and GOG-99 previously established that EBRT reduces locoregional recurrence after primary surgery of high intermediate risk endometrial cancer [28,30,31]. Brachytherapy was then established as a noninferior option with reduced GI side effects compared to EBRT [5]. Other studies have used brachytherapy as a boost in combination with EBRT for higher-stage endometrial cancer [32]. Thus, brachytherapy has demonstrated to be a significant treatment modality in reducing the recurrence of endometrial cancer. These landmark trials predominantly included White women, which, in general, calls into question the applicability of the data to other races particularly those of heterogenous socioeconomic status. Proponents of health equity within clinical trials advocate for “overrepresentation” of patients from minority population in future clinical trials, as merely meeting equal representation for minoritized population does not contribute to advancements in health equity or inclusion [33].

For locoregional cervical cancer, timely brachytherapy within 8 weeks of EBRT with cisplatin-based chemotherapy has shown to significantly improve survival as demonstrated by multiple retrospective analyses [8,9]. Our data demonstrate that there are no significant differences in time to radiation across the racial groups, suggesting adherence to the treatment timing recommendations made in the 1990s [8]. This is in the context that several studies observe a decline in brachytherapy use and attribute it to the introduction of intensity modulation radiation therapy (IMRT) and stereotactic body radiation therapy (SBRT) use over brachytherapy [6,34]. While there have not been clear randomized control trials to establish the efficacy of SBRT and IMRT over brachytherapy with respect to locoregional control for cervical cancer, the popularity of these EBRT modalities may be due to the intensive technical requirements of brachytherapy (e.g., necessity of procedure room time and space, anesthesia availability, clinical staffing). This difference in resource requirement for brachytherapy may explain in part why women in our study treated at community cancer centers, as defined by the Commission on Cancer, were associated with the decreased likelihood treatment with brachytherapy among Black women with cervical cancer and NHPI women with endometrial cancer. Brachytherapy is operator dependent, akin to other technical procedures, with clinical outcomes generally dependent on the overall experience and comfort of the treating provider. A lack of exposure and comfort may lead to inaccurate placement and setup of the brachytherapy applicators, which is known to be associated with inferior local control [6,35].

Understanding the intersection of race and oncologic outcomes is an underpinning of cancer disparities research, which exposes populations that may be receiving inequitable care. Recent studies have shown populations less likely to receive brachytherapy include Non-Hispanic Black patients, the uninsured, and those with Medicaid and Medicare insurance [7,9]. Another investigation found that receiving brachytherapy was associated with improved survival, even if not received within the 8 weeks [9]. This was further verified by the observance that when Black patients did receive brachytherapy, there was no difference in survival by race [9,12,36]. Notably, none of these studies included the NHPI race [21,22]. In order to truly understand racial disparities in oncology research, all racial categories as federally defined by the United States should be included in studies taking place in the United States, particularly with large database studies with the sample size and power to do so [23].

Moreover, there are many recent and ongoing studies in gynecologic malignancies evaluating the utility of select molecular markers in the personalization and optimization of adjuvant therapy since the updated 2020 WHO diagnostic classification of endometrial cancer [37,38,39,40]. Clinical trials such as PORTEC-4a and RAINBO aim to evaluate how molecular-directed adjuvant treatment strategies can be optimized when stratified using p53 mutation, mismatch repair deficiency, and POLE mutation status [37,39]. While the NCDB does not comprehensively cover these mutational statuses in this study, future studies are warranted to investigate which racial or ethnic groups, if any, may be enriched with these molecular markers to further fine tune adjuvant treatment options.

Access to appropriate care is a modifiable risk factor that may be contributing to racial disparities seen in cervical cancer treatment. Non-Hispanic Black patients have been shown previously to receive more RT, but not the appropriate initial surgery, for stage I disease [41]. They also found that Non-Hispanic Black women were treated less with brachytherapy for more advanced stage disease. In yet another study using the California Cancer Registry, researchers found only 45% of eligible patients with locoregional cervical cancer received brachytherapy [20]. They found that women who did not receive brachytherapy, who were characteristically from lower socioeconomic, Non-Hispanic Black race/ethnicity, and over the age of 80 years, ultimately had a decreased cause-specific and overall survival [20]. In our study, rates of brachytherapy underutilization were similar.

The strengths of this study included the large sample size of over 150,000 women recorded in the real-world database used in this analysis. While most studies are unable to capture United States Indigenous populations (American Indian, Alaska Native, Native Hawaiian, and other Pacific Islanders), the NCDB is comprehensive enough to include these historically marginalized groups into the multivariable logistic regressions [22,23,42]. The inclusion criteria also were a strength of the study as the patients evaluated were selected based on clinical trial inclusion criteria known to benefit from treatment with brachytherapy. This therefore limited the bias of the analysis in comparison to a study design that would have included women with lower stage disease who may not have needed adjuvant EBRT or brachytherapy.

There are limitations to the study given its retrospective nature and source of data used. While the NCDB captures over 70% of cancer diagnoses in the United States, patients captured are hospital-representative rather than population-representative. Moreover, multiracial individuals are not captured well in this database, and thus some multiracial individuals may be missing from the dataset, which possibly limits the full context of race-based disparities. Regarding the reference group, while Non-Hispanic White women were used as the referent control due to their representative majority in the dataset, this is not to imply that Non-Hispanic White women should be viewed as the “standard” by which women from other races should be compared to. This study focused on the five federally defined categories of race in the United States; further studies will be needed to elucidate differences in the context of Hispanic race, which may have reduced the small population size of minority populations such as AI/AN or NHPI women in this study. Lastly, aside from the education, income, and rurality variables used in this study, other social determinants of health factors including key modifiable risk factors were not accounted for in the analysis, given the lack of this granular data within the NCDB. Future studies will be needed to investigate how diet, exercise, alcohol intake, and other modifiable risk factors may play into the decision to treat endometrial and cervical cancers with brachytherapy.

Our findings underscore the importance of disaggregating NHPI women from Asian women with gynecologic cancers. Despite their common aggregation, these two populations represent two unrelated groups and should not be combined per United States federal race standards. Multiple recent large retrospective studies have demonstrated that NHPI women with endometrial and cervical cancers have significantly higher mortality when compared to Asian women [26,43,44]. The present findings add new context to the treatment of gynecologic cancers among NHPI and Asian women. Differences in brachytherapy between NHPI and Asian populations may explain in part the disparities in overall survival among NHPI and Asian women with endometrial and cervical cancers [26].

## 5. Conclusions

The present study demonstrates that there are significant differences in the use of brachytherapy among women with cervical and endometrial cancers in the United States by patient race, cancer stage, and time of diagnosis. Adjusted for patient demographics, cancer characteristics, and treatment modalities, Black women are the least likely to receive brachytherapy for cervical cancer and NHPI are the least likely to receive brachytherapy for endometrial cancer. This is particularly true for women treated at community cancer centers. These findings suggest possible populations and treatment facilities that may benefit from additional resources to promote health equity among women with gynecologic cancers.

## Figures and Tables

**Table 1:** Patient demographics, cancer characteristics, and treatment modalities. Characteristics are stratified by cancer type. Statistics are presented as n (%) or median (interquartile range). Comorbidity index represents Charlson Deyo comorbidity score. Abbreviations: White = Non-Hispanic White; AI/AN = American Indian and Alaska Native; NHPI = Native Hawaiian and other Pacific Islander; academic = academic/research program; community = community cancer program; comprehensive community = comprehensive community cancer program; integrated = integrated network cancer program; LVSI = lymphovascular space invasion.

Characteristic	Overall n = 154,799	Cervical n = 13,857	Endometrial n = 140,942
Follow-up, months, median (IQR)	69 (38–111)	45 (24–86)	71 (41–112)
Age, years, median (IQR)	63 (56–71)	53 (43–63)	64 (57–72)
Race/ethnicity			
White	133,751 (86%)	9934 (72%)	123,817 (88%)
AI/AN	548 (0.4%)	100 (0.7%)	448 (0.3%)
Asian	3213 (2.1%)	534 (3.9%)	2679 (1.9%)
Black	16,841 (11%)	3243 (23%)	13,598 (9.6%)
NHPI	446 (0.3%)	46 (0.3%)	400 (0.3%)
Year diagnosis			
2004–2010	87,501 (57%)	7788 (56%)	79,713 (57%)
2011–2017	67,298 (43%)	6069 (44%)	61,229 (43%)
Distance to hospital	0.12 (0.05–0.31)	0.12 (0.05–0.30)	0.12 (0.05–0.31)
Unknown	11,421 (7.3%)	922 (6.7%)	10,499 (7.4%)
Income			
Higher income	85,218 (55%)	5971 (43%)	79,247 (56%)
Lower income	57,965 (37%)	6952 (50%)	51,013 (36%)
Missing	11,616 (7.5%)	934 (6.7%)	10,682 (7.6%)
Rural–urban/metro			
Metropolitan	124,329 (80%)	10,860 (78%)	113,469 (81%)
Urban–rural	25,395 (16%)	2623 (19%)	22,772 (16%)
Missing	5075 (3.3%)	374 (2.7%)	4701 (3.3%)
Education			
More education	83,771 (54%)	5625 (41%)	78,146 (55%)
Less education	59,486 (38%)	7302 (53%)	52,184 (37%)
Missing	11,542 (7.5%)	930 (6.7%)	10,612 (7.5%)
Insurance Status			
Private insurance	70,596 (46%)	5370 (39%)	65,226 (46%)
Medicaid/Medicare	76,849 (50%)	6729 (49%)	70,120 (50%)
Uninsured	5174 (3.3%)	1355 (9.8%)	3819 (2.7%)
Missing	2180 (1.4%)	403 (2.9%)	1777 (1.3%)
Comorbidity Index			
≤2	146,874 (95%)	13,359 (96%)	133,515 (95%)
3+	7925 (5.1%)	498 (3.6%)	7427 (5.3%)
Facility Type			
Academic	62,623 (40%)	5787 (42%)	56,836 (40%)
Community	7435 (4.8%)	751 (5.4%)	6684 (4.7%)
Comprehensive community	58,907 (38%)	3714 (27%)	55,193 (39%)
Integrated	21,650 (14%)	1345 (9.7%)	20,305 (14%)
Missing	4184 (2.7%)	2260 (16%)	1924 (1.4%)
United States Region			
West	20,929 (14%)	1423 (10%)	19,506 (14%)
Midwest	41,440 (27%)	3042 (22%)	38,398 (27%)
Northeast	34,603 (22%)	2407 (17%)	32,196 (23%)
South	53,643 (35%)	4725 (34%)	48,918 (35%)
Missing	4184 (2.7%)	2260 (16%)	1924 (1.4%)
Grade			
1	41,179 (27%)	947 (6.8%)	40,232 (29%)
2	69,647 (45%)	6890 (50%)	62,757 (45%)
3	43,973 (28%)	6020 (43%)	37,953 (27%)
Stage			
IA	37,908 (24%)	96 (0.7%)	37,812 (27%)
IB	65,074 (42%)	2045 (15%)	63,029 (45%)
II	19,038 (12%)	4910 (35%)	14,128 (10%)
III	31,363 (20%)	6094 (44%)	25,269 (18%)
IVA	1416 (0.9%)	712 (5.1%)	704 (0.5%)
Histology			
Adenocarcinoma	12,188 (7.9%)	1296 (9.4%)	10,892 (7.7%)
Clear cell/serous	14,854 (9.6%)	86 (0.6%)	14,768 (10%)
Endometroid	115,243 (74%)	220 (1.6%)	115,023 (82%)
Squamous cell carcinoma	12,514 (8.1%)	12,255 (88%)	259 (0.2%)
LVSI	76,952 (50%)	6830 (49%)	70,122 (50%)
Deceased	39,499 (26%)	6156 (44%)	33,343 (24%)

**Table 2:** Treatment practice patterns for patients with cervical cancer receiving radiation therapy, chemotherapy, and surgery stratified by race. Statistics are presented as n (%) or median (interquartile range). Days to radiation therapy was defined by the interval of time between the date of diagnosis and the start of radiation therapy. Fisher’s exact and ANOVA tests were performed for categorical and continuous variables, respectively. Abbreviations: White = Non-Hispanic White; AI/AN = American Indian and Alaska Native; NHPI = Native Hawaiian and Other Pacific Islander; EBRT = external beam radiation therapy; RT = radiation therapy.

Characteristic	Overall n = 13,857	White n = 9934	AI/AN n = 100	Asian n = 534	Black n = 3243	NHPI n = 46
EBRT	10,796 (78%)	7784 (78%)	74 (74%)	442 (83%)	2457 (76%)	39 (85%)
Any brachytherapy	8440 (61%)	6173 (62%)	59 (59%)	336 (63%)	1842 (57%)	30 (65%)
RT modality						
Brachytherapy	2050 (15%)	1451 (15%)	14 (14%)	56 (10%)	526 (16%)	3 (6.5%)
Brachytherapy + EBRT	6390 (46%)	4722 (48%)	45 (45%)	280 (52%)	1316 (41%)	27 (59%)
EBRT	4406 (32%)	3062 (31%)	29 (29%)	162 (30%)	1141 (35%)	12 (26%)
No RT	1011 (7.3%)	699 (7.0%)	12 (12%)	36 (6.7%)	260 (8.0%)	4 (8.7%)
Time to RT, days	37 (23–56)	35 (22–54)	38 (24–60)	38 (26–57)	41 (24–62)	37 (28–56)
Unknown	1026	700	9	74	235	8
Chemotherapy	11,855 (86%)	8565 (86%)	88 (88%)	457 (86%)	2706 (83%)	39 (85%)
Surgery	0 (0%)	0 (0%)	0 (0%)	0 (0%)	0 (0%)	0 (0%)

**Table 3:** Treatment practice patterns for patients with endometrial cancer receiving radiation therapy, chemotherapy, and surgery stratified by race. Statistics presented as n (%) or median (interquartile range). Days to radiation therapy was defined by the interval of time between the date of diagnosis and the start of radiation therapy. Fisher’s exact and ANOVA tests were performed for categorical and continuous variables, respectively. Abbreviations: White = Non-Hispanic White; AI/AN = American Indian and Alaska Native; NHPI = Native Hawaiian and Other Pacific Islander; EBRT = external beam radiation therapy; RT = radiation therapy.

Characteristic	Overall n = 140,942	White n = 123,817	AI/AN n = 448	Asian n = 2679	Black n = 13,598	NHPI n = 400
EBRT	26,519 (19%)	22,906 (18%)	75 (17%)	650 (24%)	2812 (21%)	76 (19%)
Any brachytherapy	35,979 (26%)	31,565 (25%)	92 (21%)	682 (25%)	3558 (26%)	82 (20%)
RT Modality						
Brachytherapy	25,559 (18%)	22,620 (18%)	65 (15%)	431 (16%)	2386 (18%)	57 (14%)
Brachytherapy + EBRT	10,420 (7.4%)	8945 (7.2%)	27 (6.0%)	251 (9.4%)	1172 (8.6%)	25 (6.2%)
EBRT	16,099 (11%)	13,961 (11%)	48 (11%)	399 (15%)	1640 (12%)	51 (13%)
No RT	88,864 (63%)	78,291 (63%)	308 (69%)	1598 (60%)	8400 (62%)	267 (67%)
Time to RT, days	94 (70–134)	92 (69–131)	95 (70–168)	102 (75–158)	109 (78–160)	104 (70–152)
Unknown	89,755	79,038	309	1682	8448	278
Chemotherapy	27,888 (20%)	23,143 (19%)	94 (21%)	722 (27%)	3821 (28%)	108 (27%)
Surgery	140,942 (100%)	123,817 (100%)	448 (100%)	2679 (100%)	13,598 (100%)	400 (100%)

## Data Availability

Data from the National Cancer Database are available in a publicly accessible repository.
